# Radiomics and Its Applications and Progress in Pancreatitis: A Current State of the Art Review

**DOI:** 10.3389/fmed.2022.922299

**Published:** 2022-06-23

**Authors:** Gaowu Yan, Gaowen Yan, Hongwei Li, Hongwei Liang, Chen Peng, Anup Bhetuwal, Morgan A. McClure, Yongmei Li, Guoqing Yang, Yong Li, Linwei Zhao, Xiaoping Fan

**Affiliations:** ^1^Department of Radiology, Suining Central Hospital, Suining, China; ^2^Department of Radiology, The First Hospital of Suining, Suining, China; ^3^Department of Radiology, The Third Hospital of Mianyang and Sichuan Mental Health Center, Mianyang, China; ^4^Department of Radiology, The First Affiliated Hospital of Chongqing Medical University, Chongqing, China; ^5^Department of Gastroenterology, The First Hospital of Suining, Suining, China; ^6^Sichuan Key Laboratory of Medical Imaging, Department of Radiology, Affiliated Hospital of North Sichuan Medical College, Nanchong, China; ^7^Department of Radiology and Imaging, Institute of Rehabilitation and Development of Brain Function, The Second Clinical Medical College of North Sichuan Medical College, Nanchong Central Hospital, Nanchong, China

**Keywords:** radiomics, acute pancreatitis, chronic pancreatitis, autoimmune pancreatitis, pancreatic ductal adenocarcinoma, computed tomography, magnetic resonance imaging, positron emission tomography/computed tomography

## Abstract

Radiomics involves high-throughput extraction and analysis of quantitative information from medical images. Since it was proposed in 2012, there are some publications on the application of radiomics for (1) predicting recurrent acute pancreatitis (RAP), clinical severity of acute pancreatitis (AP), and extrapancreatic necrosis in AP; (2) differentiating mass-forming chronic pancreatitis (MFCP) from pancreatic ductal adenocarcinoma (PDAC), focal autoimmune pancreatitis (AIP) from PDAC, and functional abdominal pain (functional gastrointestinal diseases) from RAP and chronic pancreatitis (CP); and (3) identifying CP and normal pancreas, and CP risk factors and complications. In this review, we aim to systematically summarize the applications and progress of radiomics in pancreatitis and it associated situations, so as to provide reference for related research.

## Introduction

### Radiomics and Its Process

Inspired by the knowledge systems and research fields of such as genomics, proteomics, radiogenomics, etc., Lambin et al. first proposed the concept of radiomics in 2012 (1–6). Radiomics refers to high-throughput extraction and analysis of a large number of advanced quantitative imaging features from medical images obtained by computed tomography (CT), magnetic resonance imaging (MRI) or positron emission tomography (PET) (2). The workflow of radiomics mainly includes the following steps (1–6). (1) *Image acquisition* is the first step of radiomics. The images may come from CT, MRI, PET, as well as X-ray radiography and ultrasonography (US), etc. (7–10). Because the distribution of images features may be affected by many factors such as equipment vendors, scanning protocols, imaging parameters, reconstruction algorithms, etc., it is of great importance to establish standards and consensus imaging protocols. (2) *Image segmentation* uses dedicated software to draw two dimensions (2-D) or three dimensions (3-D) of regions of interest (ROIs) of lesions or organs by means of manual, semi-automatic, or automatic segmentations.

(3) *Image preprocessing* is to homogenize the data before extracting radiomics features which mainly includes two methods: image resampling and gray-level discretization (4). *Features extraction* uses dedicated software or software packages to extract morphological features, first-order statistical features, second-order statistical features, and high-order statistical features from 2-D or 3-D ROIs after segmentation. Morphological features (*n* = 16) are used to describe the 3-D shape and size of a ROI including asphericity, compactness, maximum diameter, sphericity, surface area, surface to volume ratio, volume, etc. The first-order statistical feature (*n* = 18) represents the histogram of voxel intensity values contained within a ROI to include mean, median, maximum, minimum, standard deviation, percentile, skewness, kurtosis, uniformity, energy, entropy, etc. Second order statistical features are used to describe the spatial distribution of voxel intensities within a ROI to include gray-level co-occurrence matrix (GLCM), gray-level run-length matrix (GLRLM), gray-level size-zone matrix (GLSZM), gray-level distance-zone matrix (GLDZM), neighborhood gray tone difference matrix (NGTDM), and neighboring gray level dependence (NGLDM). After applying filters or mathematical transformations to the images, the higher-order statistics features can be obtained (5). *Feature selection* is the process of removing redundant features and selecting the most relevant features according to specific research tasks. Common methods are univariate analysis, logistic regression analysis, least absolute shrinkage and selection operator (LASSO), minimum redundancy maximum relevance (MRMR), etc. (6). *Modelization and validation* is after a classification or prediction model is established, it needs to be tested internally and externally to evaluate the robustness and repeatability of the model.

In recent years, due to the progress and rapid developments of various hardware and software technologies, radiomics has gradually developed into a relatively mature discipline or medical image analysis method (1). There are more and more publications on the application of radiomics for the diagnosis, differential diagnosis, treatment options, and prognosis evaluation of many human diseases (11–16). Among them, Hong et al. (13) extracted 10 radiomics features from the contrast-enhanced CT (CECT) images of 241 patients with a bone island or osteoblastic metastasis to establish a random forest (RF) prediction model. The results showed that the RF model based on CT was helpful to differentiate bone islands from osteoblastic metastases, and its diagnostic performance was higher than that of inexperienced radiologists but equivalent to that of experienced radiologists. In another study, Tian et al. (16) reported the diagnostic value of preoperative evaluation of microvascular invasion of solitary small hepatocellular carcinoma (HCC) based on nomogram of gadolinium ethoxybenzyl diethylenetriamine pentaacetic acid (Gd-EOB-DTPA) enhanced MRI. The results indicated that the clinical-radiological-radiomics model achieved the highest diagnostic performance with area under the receiver operating characteristic curves (AUCs) of 0.934, 0.889 and 0.875 for the training, internal and external validation sets, respectively.

In this review, we aim to systematically summarize the applications and progress of radiomics in pancreatitis and associated situations (Table 1) so as to provide reference for related research.

**Table 1:** Characteristics of the included publications on radiomics in pancreatitis.

**Study ID**	**Year**	**Country**	**Design**	**Sample size**	**Objective (s)**	**Reference standard**	**Imaging modality**	**Imaging phases (slice thickness)**	**Segmentation method**	**Segmentation software**	**Feature extraction software**	**Feature type**
Chen et al. (17)	2019	China	Retrospective	389	Predicting the recurrence of AP	Follow-up	Somatom Definition AS and Somatom Definition Flash (Siemens Healthineers), and LightSpeed VCT (GE Healthcare)	Arterial phase and venous phase images (5.0 mm)	Manual	IBEX	IBEX	S and Q
Hu et al. (18)	2022	China	Retrospective	190	Predicting the recurrence of AP	Follow-up	3.0 T MRI (Discovery 750, GE Healthcare)	T2WI (5.0 mm)	Manual	IBEX	IBEX	S and Q
Lin et al. (19)	2020	China	Retrospective	259	Predicting severity of AP	2012 revised Atlanta classification of AP	3.0T MRI (Discovery 750, GE Healthcare)	Portal venous phase images (5.2 mm)	Manual	IBEX	IBEX	S and Q
Zhou et al. (20)	2021	China	Retrospective	135	Predicting EXPN in AP	Pathology and follow-up	3.0 T MRI (Discovery 750, GE Healthcare)	T2WI images of extra pancreatic collections and late arterial phase images of the pancreatic parenchyma (6.0 mm)	Manual	IBEX	IBEX	S and Q
Zhang et al. (21)	2022	China	Retrospective	138	Differentiating MFCP from PDAC	Pathology and CP consensus	Brilliance-16P (Philips Healthcare) and Aquilion ONE (Canon Medical Systems)	Portal venous phase images	Manual	3D Slicer	Pyradiomics	S and Q
Liu et al. (22)	2022	China	Retrospective	102	Distinguishing PC from MFCP	Pathology and follow-up	3.0 T MRI (MAGNETOM Skyra, Siemens Healthineers)	Axial T1WI, T2WI, DWI (b=800 s/mm^2^), and ADC images	Manual	ITK-Snap	Pyradiomics	S and Q
Ma et al. (23)	2022	China	Retrospective	175	Differentiating between PC and CP (AIP and MFCP)	Including pathology and follow-up	Discovery CT 750 HD, Revolution CT, and Optima CT660 (GE Healthcare)	Arterial phase and venous phase images	Manual	MITK	Pyradiomics	S and Q
Deng et al. (24)	2021	China	Retrospective	119	Distinguishing PDAC from MFCP	Pathology	3.0 T MRI (Discovery 750, GE Healthcare)	Axial T1WI, T2WI, and the arterial phase and portal venous phase images	Manual	IBEX	IBEX	S and Q
Ren et al. (25)	2020	China	Retrospective	109	Differentiating MFCP from PDAC	Pathology	Brilliance 64 (Philips Healthcare) and Optima 670 (GE Healthcare)	Unenhanced CT images (3.0 mm)	Manual	ITK-SNAP	Analysis Kit	Q only
Ren et al. (26)	2019	China	Retrospective	109	Differentiating MFCP from PDAC	Pathology	Brilliance 64 (Philips Healthcare) and Optima 670 (GE Healthcare)	Arterial and portal phase CT images (3.0 mm)	Manual	ITK-SNAP	Analysis Kit	S and Q
Zhang et al. (27)	2019	China	Retrospective	109	Differentiating MFCP from PDAC	Pathology	Brilliance 64 (Philips Healthcare), Light speed VCT and Discovery HD750 (GE Healthcare)	Parenchymal phase images (5.0 mm)	Manual	ITK-SNAP	Analysis Kit	S and Q
Li et al. (28)	2022	China	Retrospective	97	Differentiating AIP from PDAC	Pathology and follow-up	Brilliance-16P (Philips Healthcare); Aquilion ONE (Canon Medical Systems)	Portal venous phase images (0.8/1.0 mm)	Manual	3D Slicer	Pyradiomics	S and Q
Liu et al. (29)	2021	China	Retrospective	112	Differentiating AIP and PDAC	Pathology and follow-up	PET/CT (Biograph64, Siemens Healthineers)	early and delayed imaging (3.0 mm)	Manual	3D Slicer	MATLAB R2018a	S and Q
Linning et al. (30)	2020	China	Retrospective	96	Differentiating AIP and PDAC	Pathology and follow-up	A range of helical multidetector (16, 64, 128, and 256 slices)	Non-contrast, arterial, and venous phases (1.0-5.0 mm)	Manual	In-house imaging platform	In-house MATLAB 2016b program	S and Q
Park et al. (31)	2020	USA	Retrospective	182	Differentiating AIP from PDAC	Pathology and follow-up	Somatom Definition, Definition Flash, or Force, and Somatom Sensation (Siemens Healthineers)	Arterial phase and venous phase images (0.75/3.0 mm)	Manual	Velocity AI	Velocity AI	S and Q
Zhang et al. (32)	2019	China	Retrospective	111	Differentiating AIP and PDAC	Pathology and follow-up	PET/CT (Biograph64, Siemens Healthineers)	- (0.98 mm)	Manual	3D Slicer	MATLAB R2017a	Q only
Zhang et al. (33)	2019	China	Retrospective	111	Differentiating AIP and PDAC	Pathology and follow-up	PET/CT (Biograph64, Siemens Healthineers)	- (0.6 mm)	Manual	3D Slicer	MATLAB R2017a	S and Q
Mashayekhi et al. (34)	2020	USA	Retrospective	56	Differentiating FAP, RAP, and CP	Clinical criteria	Including Sensation 64 (Siemens Healthineers)	Portal venous phase images (3 mm)	Manual	In-house MATLAB program	In-house MATLAB program	Q only
Frøkjær et al. (35)	2020	Denmark	Retrospective	99	Differentiating CP from healthy pancreas; classification of CP based on two risk factors and two complications	Lüneburg criteria	1.5T MRI (Signa HDxt, GE Healthcare)	DWI (b = 0 s/mm^2^) (2.6 mm)	Manual	3D Slicer	Pyradiomics	Q only
**Study ID**		**Type of extracted features**		**Number of extracted features**	**Number of statistically significant features**	**Feature reduction and classification method**	**Modeling method**	**Evaluation index**	**Main conclusions**		**%RQS (points)**	
Chen et al. (17)		Shape features; First-order texture features; Second-order texture features		412	10 (five from arterial phase and five from portal phase)	Independent samples *t*-test, Mann-Whitney *U* test, LASSO regression, and Spearman correlation	Multivariable logistic regression analysis and SVM	ROC curve analysis for radiomics and clinical models	The radiomics model based on CECT performed well in predicting AP recurrence		16 (44%)	
Hu et al. (18)		Shape features; First-order texture features; Second-order texture features		513	4	LASSO	Multivariable logistic regression analysis	ROC curve analysis for radiomics, clinical, and combined models	Radiomics features based on MRI-T2WI could be used as biomarkers to predict the recurrence of AP		12 (33%)	
Lin et al. (19)		Shape features; First-order texture features; Second-order texture features		353	11	Independent sample *t*-test, Mann–Whitney *U* test, and Boruta algorithm	SVM	ROC curve analysis for radiomics model, and scoring systems of APACHE II, BISAP and MRSI	CEMRI based radiomics model had good performance in the early prediction of AP severity		15 (42%)	
Zhou et al. (20)		Shape features; First-order texture features; Second-order texture features		350	22 (12 from the extrapancreatic collection images and 10 from the pancreatic parenchyma images)	Independent sample *t*-test, Mann–Whitney *U* test, and LASSO	SVM	ROC curve analysis for radiomics models, clinical model, and scoring systems of EPIM and MRSI	The MRI-based radiomics models of both the extrapancreatic collections and the pancreatic parenchyma had excellent predictive performance for early EXPN		16 (44%)	
Zhang et al. (21)		Shape features; First-order texture features; Second-order texture features		1,409	8	Variance analysis, Spearman's correlation analysis, and LASSO	Multivariable logistic regression analysis	ROC curve analysis for the CT model and radiomics models	The CT and radiomics models both were shown to be reasonably accurate in their differentiation of MFCP from PDAC in patients with CP		15 (42%)	
Liu et al. (22)		Shape features; First-order texture features; Second-order texture features		960	6 (1 from T1WI, 2 from T2WI, 1 from DWI, and 2 from ADC maps)	MRMR and LASSO algorithms	Nomogram of the mixed model incorporating the radiomic signature, the CA19–9 level, and the CEA level	Individual T1WI, T2WI, DWI, and ADC models; clinical model; multiparametric MRI model; mixed-prediction model	A comprehensive model based on multiparametric MRI and clinically independent risk factors displayed the best evaluation performance		16 (44%)	
Ma et al. (23)		Shape features; First-order texture features; Second-order texture features		1,037	2 (both from venous phase CT images)	Preserve features with good consistence, univariate Wilcoxon rank–sum test, correlation analysis, LASSO	Multivariable logistic regression analysis	ROC curve analysis for the arterial phase, venous phase, and arterial phase combined with venous phase radiomics model; clinical feature model; radiomics combined with clinical feature comprehensive model	The radiomics combined with clinical feature model could be a potential tool to distinguish PC from CP		16 (44%)	
Deng et al. (24)		First-order texture features; Second-order texture features		410	28 (the number of included features in the T1WI, T2WI, arterial phase and portal venous phase feature subsets were 5, 7, 7, and 9, respectively)	Independent sample *t*-test, Mann–Whitney *U* test, LASSO	SVM	ROC curve analysis for T1WI, T2WI, and the arterial phase and portal venous phase radiomics models, and a clinical model	Radiomic models based on multiparametric MRI have the potential to distinguish PDAC from MFCP		17 (47%)	
Ren et al. (25)		Shape features; First-order texture features; Second-order texture features		396	10	Mann–Whitney *U* test and MRMR	RF	ROC curve analysis for radiomics model	Unenhanced CT texture analysis can be a promising non-invasive method in discriminating MFCP from PDAC		10 (28%)	
Ren et al. (26)		Shape features; First-order texture features; Second-order texture features		396	9 (five were arterial phase texture parameters and four portal phase texture parameters)	Mann–Whitney *U* test and MRMR	Multivariate logistic regression analysis	ROC curve analysis for imaging feature-based, texture feature-based models in arterial phase, and portal phase, and the combined model	CT texture analysis demonstrates great potential to differentiate MFCP from PDAC		10 (28%)	
Zhang et al. (27)		First-order texture features; Second-order texture features		160	4	LASSO	Multivariate logistic regression analysis	ROC curve analysis for imaging feature-based, texture feature-based models in parenchymal phase, and the combined model	The CECT combined with texture analysis model has the best diagnostic efficiency for differentiating MFCP from PDAC		10 (28%)	
Li et al. (28)		Shape features; First-order texture features; Second-order texture features		1,409	4 (from portal venous phase CT images	Variance analysis, Spearman's correlation analysis, and LASSO	Radiomics score	ROC curve analysis for radiomics score	The portal rad-score can accurately and non-invasively differentiate fAIP from PDAC		10 (28%)	
Liu et al. (29)		Shape features; First-order texture features; Second-order texture features; MIP features		514	10 (three from CT, four from PET-early, and three from PET-delay)	SVM-RFE	SVM-LKF	ROC curve analysis for fusion feature based model, dual-time PET/CT images radiomics model and clinical diagnostic indicators based model	The radiomics model based on ^18^F-FDG PET/CT dual-time images provided promising performance for discriminating AIP from PDAC		15 (42%)	
Linning et al. (30)		Shape features; First-order texture features; Second-order texture features		1,160	18 (six from non-contrast, arterial, and venous phases, respectively)	Unsupervised hierarchical clustering, MRMR, and IFS	RF	ROC curve analysis for the non-contrast, arterial phase, venous phase, and hybrid of three phases radiomics models	Radiomics is helpful for a differential diagnosis of AIP in clinical practice as a non-invasive and quantitative method		9 (25%)	
Park et al. (31)		Shape features; First-order texture features; Second-order texture features; Filtered image features		431	35	MRMR	RF	ROC curve analysis for the arterial phase and venous phase radiomics features	Radiomic features help differentiate AIP from PDAC		8 (22%)	
Zhang et al. (32)		First-order texture features; Second-order texture features; Filtered image features		418	8	Fisher's criterion >0.01 and SFS	SVM	ROC curve analysis for different feature selection and classification methods	The results proved that texture analysis of lesions helps to achieve accurate differentiation of AIP and PDAC		13 (36%)	
Zhang et al. (33)		Shape features; First-order texture features; Second-order texture features		251	10	Spearman correlation, MRMR, and SVM	RF, adaptive boosting, and SVM	ROC curve analysis for different feature selection and classification methods	Radiomics could aid the non-invasive differentiation of AIP and PDAC in 18F-FDG PET/CT images and the integration of multi-domain features is beneficial for the differentiation		15 (42%)	
Mashayekhi et al. (34)		Shape features; First-order texture features; Second-order texture features		54	11	Wilcoxon rank-sum test	Isomap and SVM	ROC curve analysis for radiomic features	Certain radiomic features on CT imaging can differentiate patients with FAP, RAP, and CP		10 (28%)	
Frøkjær et al. (35)		Shape features; First-order texture features; Second-order texture features; Filtered image features		851	5 (for differentiation between healthy pancreas and CP)	10-fold cross-validation forward selection procedure	Naive Bayes classifier	The average m-fold performance metrics for five classifiers	Pancreatic texture analysis demonstrated to be feasible in patients with CP and discriminate clinically relevant subgroups based on etiological risk factors and complications		8 (22%)	

*AP, acute pancreatitis; RAP, recurrent acute pancreatitis; CP, chronic pancreatitis; MFCP, mass-forming chronic pancreatitis; AIP, autoimmune pancreatitis; fAIP, focal type autoimmune pancreatitis; PC, pancreatic cancer; PDAC, pancreatic ductal adenocarcinoma; EPXN, extrapancreatic necrosis; FAP, functional abdominal pain; CT, computed tomography; CECT, contrast-enhanced computed tomography; MRI, magnetic resonance imaging; CEMRI, contrast-enhanced MRI; T1WI, T1-weighted imaging; T2WI, T2-weighted imaging; DWI, diffusion weighted imaging; ADC, apparent diffusion coefficient; EUS, endoscopic ultrasound; ^18^F-FDG PET/CT, ^18^F-fluorodeoxglucose positron emission tomography/computed tomography; CAD, computer-aided diagnosis; MIP, maximum intensity projection; MRMR, minimum-redundancy maximum-relevance; LASSO, least absolute shrinkage and selection operator; SFS, sequential forward selection; IFS, incremental forward search; RF, random forest; SVM, support vector machine; SVM-RFE, support vector machine recursive feature elimination; SVM-LKF, support vector machine with a linear kernel function; MITK, medical imaging interaction toolkit; S, semantic; Q, quantitative; RQS, radiomics quality score; ROC, receiver operating characteristic curve; NA, not available; EPIM, extrapancreatic inflammation on MRI; MRSI, magnetic resonance severity index; APACHE II, acute physiology and chronic health evaluation II; BISAP, bedside index for severity in acute pancreatitis*.

## Clinical Applications

### Predicting Recurrent Acute Pancreatitis

Acute pancreatitis (AP) is a common disease in clinical practice and meta-analysis showed that the annual incidence rate of AP in the world is about 33.74/100 000, along with an annual mortality rate of about 1.16/100 000 (36). With the increase in population aging, biliary calculus, hyperlipidemia, obesity, and many other AP risk factors, the incidence of AP is also gradually increasing (37–39). Recurrent acute pancreatitis (RAP) is a special type of pancreatitis, and it is different from AP and chronic pancreatitis (CP). The definition of RAP is that patients should experience at least two separate episodes of AP at least 3 months apart, and there are no abnormities in pancreatic tissue structure or function in remission (40). It is reported that the recurrence rate of AP is about 10–30% (17). About 10% of patients with first-episode of AP and 36% of patients with RAP may progress to CP, and the risk is higher among men, smokers, and alcoholics (41). Another study also reported that CP may increase the risk of pancreatic cancer (PC) in patients (42). After 5 and 9 years of the diagnosis of CP, the risk of PC in CP patients increased by eight times and three times, respectively. Therefore, early prediction of RAP and appropriate management measures can not only decrease the recurrence of AP, but it also prevents or delays its progression to CP and even PC.

Chen et al. (17) included 389 first-episode AP patients. On the CT images of arterial and venous phases, 412 radiomics features were extracted from the ROIs of the whole pancreatic parenchyma, and 10 features were finally selected to establish the prediction model. In the training cohort (*n* = 271, including 145 patients with AP and 126 patients with RAP), the sensitivity, specificity, positive predictive value (PPV), negative predictive value (NPV), accuracy and AUC of the radiomics model in predicting patients with RAP were 86.7%, 87.6%, 89.7%, 84.1%, 87.1%, and 0.941%, respectively. In the validation cohort (*n* = 118, including 63 patients with AP and 55 patients with RAP), the same diagnostic indexes of the radiomics model in predicting patients with RAP were 83.8%, 97.7%, 98.4%, 78.2%, 89.0%, and 0.929%, respectively. The results in the training and validation cohorts were all significantly higher than those of the clinical model (all *P*-values < 0.05).

Quantitative investigation on predicting RAP is still in a paucity at present. Previous studies mostly focused on the risk factors of RAP after the first attack of AP such as demography (like gender, age, etc.), and clinical characteristics (like etiology, local complications, etc.) (43–45). Chen et al. (17) first showed that the radiomics model based on CECT exhibits promising value in the early prediction of RAP. In another similar study, Hu et al. (18) constructed a multivariate logistic regression radiomics model, radiomics, and clinical characteristics combined model based on MRI-T2WI, and their results were consistent with those of Chen et al. (17).

### Predicting Clinical Severity of AP

Based on the 2012 revised Atlanta classification and definition (2012-RACD) by international consensus, AP can be divided into three categories stratified by its clinical severity: mild acute pancreatitis (MAP), moderately severe acute pancreatitis (MSAP), and severe acute pancreatitis (SAP) (46). MAP is characterized by no organ failure and local or systemic complications. It can return to normal within 1–2 weeks. Usually, there is no need for an imaging examination of the pancreas, and the mortality rate is very low. MSAP is characterized by transient organ failure (<48 h), or accompanied by local or systemic complications, while no persistent organ failure (more than 48 h) exists. MSAP can be cured without intervention or may require long-term specialist care. The mortality rate of MSAP is much lower than that of SAP. SAP is characterized by persistent single or multiple organ failure (more than 48 h). Patients with persistent organ failure usually have one or more local complications. In the first few days after AP onset, patients with persistent organ failure have an increased risk of death, and the mortality reported in the literature is as high as 36–50% (46), and the mortality rate of patients with persistent organ failure complicated with infectious necrosis is very high (46). Therefore, early prediction of the clinical severity of AP is of utmost importance, which is not only good for the early diagnosis and treatment of MSAP and SAP patients, and also in favor of the early diversion or referral of MSAP and SAP patients.

Currently, methods of early predicting the clinical severity of AP mainly depend on clinical characteristics [such as scoring systems of acute physiology and chronic health evaluation II (APACHE II, ≥eight points), bedside index for severity in acute pancreatitis (BISAP, ≥three points), Ranson (≥three points) and modified Marshall score (≥two points)], laboratory tests [such as C-reactive protein concentration (≥150 mg/l), serum procalcitonin (>0.5 ng/ml), interleukin-6 (>50 pg/l) and neutrophil/lymphocyte ratio (>10)] as well as findings on imaging examinations [such as computed tomography severity index (CTSI, ≥four points), modified computed tomography severity index (mCTSI, ≥four points), and extrapancreatic inflammation on computed tomography (EPIC, ≥four points)] (47–50).

Lin et al. (19) first reported a contrast-enhanced MRI (CEMRI) based radiomics model to predict the clinical severity of AP (MAP *vs*. MSAP and SAP). In their study, they included 259 AP patients into the training (*n* = 180, with 99 MAP and 81 MSAP and SAP patients) and validation cohorts (*n* = 79, with 43 MAP and 36 MSAP and SAP patients). From the portal vein phase images, Lin et al. (19) extracted 353 radiomics features from the ROIs that contained the whole pancreatic parenchyma, and finally they selected 11 features to establish the support vector machine (SVM) model. In the training cohort, the sensitivity, specificity, PPV, NPV, accuracy, and AUC of the radiomics model to distinguish MAP from MSAP or SAP patients were 77.8%, 91.9%, 88.7%, 83.5%, 85.6%, and 0.917%, respectively. In the validation cohort, the corresponding diagnostic indexes of the radiomics model in distinguishing MAP from MSAP or SAP patients were 75.0%, 86.0%, 81.8%, 80.4%, 81.0%, and 0.848%, respectively. The both AUCs were significantly higher than that of APACHE II, BISAP, and MRSI scoring systems (all *P*-values were < 0.05). This study showed that when compared with some existing clinical and radiological scoring systems, the portal phase MRI radiomics model may be more accurate in early predicting the clinical severity of AP.

### Predicting Extrapancreatic Necrosis in AP

Based on the 2012-RACD (46), AP can be divided into two categories according to its morphological manifestations on imaging examination: (1) interstitial edematous pancreatitis (IEP; about 85%); and (2) necrotizing pancreatitis (NP; about 15%). Based on the distribution and location of necrosis, NP can be further subdivided into three subtypes (46): (1) combined pancreatic and peripancreatic necrosis (about 75.0%); (2) peripancreatic necrosis only (about 20.0%); and (3) pancreatic necrosis only (about 5.0%). The literature indicates that compared with NP, the mortality rate of IEP is about 3.0% while the mortality rate of NP is about 17%; and if combined with infection, the mortality rate of NP can rise to about 30% (46, 51). Consequently, it is of great clinical significance to distinguish IEP from NP for predicting the prognosis of AP patients. In the international structured reporting template of AP based on CECT published in 2020, experts also highlighted the importance of radiologists to clarify the morphologic subtypes of AP, the degree and anatomic area involvement of NP, the type and location of peripancreatic collections, and some other key points in the CT reports (51).

Zhou et al. (20) used an MRI based radiomics model to predict early extrapancreatic necrosis (EXPN) in patients with AP. They enrolled 135 AP patients who were divided into the training (*n* = 94, with 47 EXPN and 47 APFC patients) and validation cohorts (*n* = 41, with 20 EXPN and 21 APFC patients). On the T2WI and late arterial phase images, Zhou et al. (20) extracted 350 image radiomics features from ROI of the peripancreatic collections (T2WI) and entire pancreatic parenchyma (late arterial phase). After dimension reduction and feature selection, 22 features (12 from the T2WI and 10 from the late arterial phase images) were selected for establishing SVM model. In the training cohort, the sensitivity, specificity, PPV, NPV, accuracy, and AUC of the T2WI peripancreatic collections and late arterial phase pancreatic parenchyma radiomics models for predicting EXPN were 97.9% and 87.2%, 85.1% and 87.2%, 86.8% and 87.2%, 97.6% and 87.2%, 91.5% and 87.2%, 0.969% and 0.931%, respectively. In the validation cohort, the corresponding diagnostic parameters of the T2WI peripancreatic collections and late arterial phase pancreatic parenchyma radiomics models for predicting EXPN were 95.0% and 75.0%, 90.5% and 90.5%, 90.5% and 88.2%, 95.0% and 79.2%, 92.7% and 82.9%, 0.976 and 0.921%, respectively. Both of the AUCs were significantly higher than those of clinical model, EPIM and MRSI scoring systems (all *P*-values < 0.05). This investigation showed that when compared with some existing clinical model and radiological scoring systems, the MRI radiomics model based on T2WI peripancreatic collections and late arterial phase pancreatic parenchyma may be able to accurately predict EXPN in AP patients at an early stage.

### Differentiating Mass-Forming Chronic Pancreatitis From Pancreatic Ductal Adenocarcinoma

Pancreatic ductal adenocarcinoma (PDAC) is a malignant tumor that originating from pancreatic ductal epithelial cells, accounting for about 80–90% of all the pancreatic cancer (PC) patients with about 60–70% of the PDACs occur in the pancreatic heads (52, 53). The prognosis of PDAC is very poor (<10%) and surgery has always been considered the first choice for the treatment of PDAC (52, 53). The mass-forming chronic pancreatitis (MFCP) is a special type of CP. Documents reported that MFCP accounts for about 27–50% of CP, and the vast majority of MFCP is located in the pancreatic heads (about 71%) (54–56). MFCP and PDAC share significant overlaps in the clinical manifestations (such as upper abdominal pain, nausea, weight loss, jaundice, diabetes, etc.), risk factors (such as alcohol, smoking, etc.), laboratory tests (such as elevated carbohydrate antigen 199 (CA199) and carcinoembryonic antigen (CEA) levels), and imaging findings (such as delayed enhancement) (57, 58). CT and endoscopic ultrasonography guided fine needle aspiration biopsy (EUS-FNA) can be used to improve the differential diagnosis accuracy of MFCP and PDAC, but both modalities are invasive examinations, which not only have sampling error, and also carry the risks of needle tract tumor seeding, bleeding, pancreatic juice leakage, etc. (59, 60). As a result, it is very difficult to accurately distinguish MFCP from PDAC prior to operation, yet it has very important clinical significance. Because accurate preoperative diagnosis of early PADC can prevent it from being resectable to unresectable, and accurate diagnosis of MFCP can avoid unnecessary surgery.

With the rapid development of medical imaging technologies, radiomics has begun to be used in the differential diagnosis of MFCP and PDAC (21–27). For example, Deng et al. (24) studied 96 patients with PDAC and 23 patients with MFCP. They extracted four sets of radiomics features from T1WI, T2WI, as well as arterial and portal phase images of MRI to establish SVM models. When compared with the clinical model based on clinical characteristics and the evaluation results of two radiologists, the results demonstrated that in the primary cohort (*n* = 64, with 51 PDAC and 13 MFCP patients), the sensitivity, specificity and AUC of T1WI, T2WI, arterial phase and portal phase radiomics models, and the clinical model were 0.961, 0.769, and 0.893; 0.941, 0.769, and 0.911; 0.961, 0.923, and 0.958; 0.980, 1.000, and 0.997; 0.529, 0.692, and 0.516, respectively. In the testing cohort (*n* = 55, with 45 PDAC and 10 MFCP patients), the corresponding diagnostic data were 1.000, 0.733, and 0.882; 0.844, 0.900, and 0.902; 0.956, 0.900, and 0.920; 0.978, 0.900, and 0.962; 0.422, 0.900, and 0.649, respectively. There were no significant differences in the diagnostic performances between the four radiomics models (all *P*-values > 0.05), but they were all better than that of the clinical model and the radiologists' evaluation (all *P*-values < 0.05). This study demonstrated that radiomics may be used to improve the differential diagnosis accuracy of MFCP and PDAC.

### Differentiating Focal Autoimmune Pancreatitis From PDAC

Autoimmune pancreatitis (AIP) is a special type of CP. Yoshida et al. first proposed the concept of AIP in 1995; and the annual incidence rate of AIP is about 3.1/100 000, accounting for about 1.9%-6.6% of CP (61, 62). Pathologically, AIP is classified into two subtypes: (1) Type I, lymphoplasmacytic sclerosing pancreatitis (LPSP); and (2) Type II, idiopathic duct-centric chronic pancreatitis (IDCP) (61, 63). At present, Type I AIP has been considered as the pancreatic manifestation of a systemic disease named IgG4-related disease (IgG4-RD) and there are now dedicated criteria for IgG4-RD and some specific organs (like pancreas, biliary tract, kidney, ophthalmic tissues, and chest) (64–67). On imaging, AIP can be manifested as diffuse AIP and focal AIP, and about 40% of Type I AIP and 85% of Type II AIP are localized (64, 65). Focal AIP overlaps obviously with PDAC in clinical manifestations (such as obstructive jaundice, epigastric pain or discomfort, weight loss, etc.) and imaging findings (focal mass in the pancreas), and an accurate differential diagnosis is very challenging. However, the treatment methods after the establishment of diagnosis are very different because AIP responds well to glucocorticoid drugs while PADC mainly needs comprehensive treatment methods such as surgery, chemotherapy and radiotherapy. Therefore, the accurate differential diagnosis of focal AIP and PDAC before the treatments has very important clinical value. Once focal AIP is misdiagnosed as PDAC, it will lead to unnecessary surgery, and once PDAC is misdiagnosed as focal AIP, it may delay the effective treatments of PDAC.

Radiomics may play a positive role in the differential diagnosis of focal AIP and PDAC (28–33). Among the studies, Linning et al. (30) studied 45 patients with focal AIP and 51 patients with PDAC to evaluate the value of radiomics model based on multi-phase CECT for the differential diagnosis of focal AIP from PDAC. The results showed that the sensitivity, specificity, PPV, NPV and accuracy of unenhanced, arterial phase, portal phase, and hybrid radiomics models were 71.11%, 86.27%, 77.19%, 82.05%, and 79.17%; 82.22%, 90.20%, 85.19%, 88.10%, and 86.46%; 93.33%, 96.08%, 92.00%, 89.13%, and 90.63%; 93.33%, 96.08%, 94.23%, 95.45%, and 94.80%, respectively. The AUCs were 0.827, 0.890, 0.953, and 0.977, respectively. The diagnostic performances were higher than those of the two radiologists (*P* < 0.05). In another study, Li et al. (28) used propensity score matching (PSM) in 45 patients with focal AIP and 51 patients with PDAC who were matched in gender, age, body mass index (BMI), and CT characteristics. They evaluated the diagnostic performance of radiomics model based on portal phase CECT images in the differential diagnosis of focal AIP and PDAC. Their results were consistent with the research of Linning et al. (30) The above two studies have shown that radiomics may play a positive role in the differential diagnosis of focal AIP and PDAC.

### Differentiating Functional Abdominal Pain, RAP, and CP

Abdominal pain is a common clinical symptom and one of the most important reasons for patients to see a doctor. Its etiologies may come from abdominal solid organs, gastrointestinal tract, biliary system, urinary system, reproductive system, chest diseases, or systemic diseases. Because abdominal pain is a non-specific clinical symptom, early identifying the causes of abdominal pain helps the clinicians and patients to choose the appropriate treatment methods. In a study, Mashayekhi et al. (34) studied 19 patients with functional abdominal pain (functional gastrointestinal diseases, FGD), 20 patients with RAP, and 17 patients with CP and explored the value of a SVM classifier based on venous phase images of CECT in distinguishing FGD, RAP, and CP. The results showed that the overall predictive accuracy of the SVM classifier was 82.1%. In the one-to-one comparison of the three groups, the sensitivity, specificity, and AUC of the FGD group were 79%, 100%, and 0.91%, respectively; the same diagnostic parameters of the RAP group were 95%, 78%, and 0.88%, respectively; while the sensitivity, specificity and AUC of the CP group were 71%, 95%, and 0.90%, respectively. The results suggested that some radiomics features may be an effective method for radiologists and gastroenterologists to distinguish FGD, RAP, and CP.

### Identifying CP and Normal Pancreas, CP Risk Factors, and Complications

Frøkjær et al. (35) studied 77 CP patients and 22 healthy controls, extracted 851 MRI texture features from diffusion-weighted imaging (DWI) images, and finally constructed five classifier models to address the potential use of MRI texture analysis of the pancreas in CP patients. The five radiomics classifiers were: (1) CP *vs*. healthy controls (with five selected radiomics features), (2) alcoholic *vs*. non-alcoholic etiology of CP (with nine selected radiomics features), (3) use of tobacco *vs*. no use of tobacco (with 10 selected radiomics features), (4) diabetes *vs*. no diabetes (with four selected radiomics features), and (5) pancreatic exocrine insufficiency *vs*. normal exocrine function (with three selected radiomics features). The results showed that the sensitivity, specificity, PPV, and accuracy of the above five radiomics classifiers were 0.71–0.97, 0.84–1.00, 0.71–1.00, and 0.82–0.98, respectively. These results implied that radiomics may be a potentially promising tool used to depict early-stage CP and monitor disease progression.

## Limitations and Solutions

Since it was proposed in 2012, due to the progress and rapid developments of various hardware and software technologies, radiomics has gradually developed into a relatively mature research field and knowledge system (1–6, 68). The authors performed a literature search in the PubMed database with the strategy of “(Radiomics [Title/Abstract]) OR (Radiomic [Title/Abstract]).” There were no restrictions on the publication time, language or research object. As of April 17, 2022, a total of 5,580 relevant publications were retrieved. This search result has proved the degree of attention paid by researchers and related fields to radiomics in the past 10 years. However, the vast majority of radiomics models reported in the current literature are still in the stage of developing research, and their clinical applications have not really been implemented. The authors believe that this phenomenon is mainly caused by the limitations of radiomics. The current radiomics research and clinical applications still have the following limitations and difficulties (69): (1) the standardization of medical imaging data is insufficient; (2) the generalization ability of the models is not good enough; (3) poor biological interpretability; and (4) the clinical utility of the models needing to be improved.

### Standardization of Medical Image Data

Standardized, homogeneous, and high-quality training data is an important cornerstone of radiomics research and clinical applications. Radiomics may refer to the FAIR guiding principles for scientific data management and stewardship that were proposed by the international community named Force 11 (The Future of Research Communications and e-Scholarship 2011) in 2016 (70). This international community emphasizes that scientific data management and stewardship should follow the principles of Findable (F), Accessible (a), Interoperable (I), and Reusable (R).

### Generalization of the Models

The performance of a radiomics model in similar and different distribution of datasets (such as various times, treatment plans, geographical locations, etc.) is called the generalization of a radiomics model. That is to say the reproducibility and transferability of a radiomics model (71) which is an important premise for the clinical applications of radiomics. It is also an important problem that needs to be solved urgently in radiomics (72, 73). In addition to increasing the data sample size and data diversity, full-automatic and semi-automatic image segmentation methods need to be advocated, and reasonable features selection and dimensionality reduction methods also need to be adopted (69, 74). Federated machine learning is also expected to provide effective solutions to the above difficulties (75, 76).

### Biological Interpretability

Radiomics researchers hope to explore the relationships between certain features and some diseases or clinical endpoints (such as the diagnosis and differential diagnosis of diseases, options of treatment schemes, predictions of treatment effects, pathological classification and grading, gene and protein phenotypes, etc.) by quantitatively extracting and analyzing image information (features) that cannot be recognized by human naked eye. This will provide more help for clinicians and patients for disease diagnosis and treatments. However, the biological interpretability of radiomics is still lacking, and the potential biological significance of each features is still unclear, which seriously hinders its clinical applications (77–79). Therefore, how can we improve the biological interpretability of radiomics is an important problem to be faced in this field.

### Clinical Utility

Radiomics models or systems with characteristics of easy to operate, short learning curve, good user experience, fast running speed, and broad use scenarios are often more in line with clinicians' work habits (80). Applications developed for mobile phones and internet users may become an effective carrier for the clinical applications of radiomics models or systems in the future.

## Conclusions and Future Perspectives

Since it was proposed in 2012, radiomics has begun to demonstrate a promising potential both in scientific research and in clinical applications, such as predicting RAP, clinical severity of AP and EXPN of AP, and differentiating MFCP and focal AIP from PDAC (Table 1). However, most of the published studies hold the limitations of a single-center, retrospective, limited sample size, and low radiomics quality score (RQS) (4). In looking forward to the future, researchers may successively report some multicenter, prospective, large sample size, and high RQS studies. In addition to these, predicting AP clinical outcomes of organ failure, infection, death, hospitalization, admission to intensive care unit (ICU) and invasive intervention; quantifying pancreatic exocrine or (and) endocrine insufficiency; predicting the possibility of AP progress to CP or CP progress to PC; and effectively combining deep learning or some other technologies with radiomics may become the potential directions (81–87).

## Author Contributions

GaowuY, GaoweY, HLi, HLia, and CP designed the study and the drafting of the paper. GaowuY, GuY, YL, and YML revised the paper critically for intellectual content. All authors participated in the literature search and data collection. All authors approved the final version of the paper to be published.

## Funding

This project was supported by grants from the Sichuan Provincial Commission of Health (Grant Nos. 18PJ138, 19PJ283, 19PJ284, and 20PJ284), Sichuan Provincial Department of Science and Technology (Grant No. 2019YFQ0028), and Science and Technology Association of Suining City (Grant Nos. 6 and 10).

## Conflict of Interest

The authors declare that the research was conducted in the absence of any commercial or financial relationships that could be construed as a potential conflict of interest.

## Publisher's Note

All claims expressed in this article are solely those of the authors and do not necessarily represent those of their affiliated organizations, or those of the publisher, the editors and the reviewers. Any product that may be evaluated in this article, or claim that may be made by its manufacturer, is not guaranteed or endorsed by the publisher.
